# A critical appraisal of guidelines and recommendations for the management of ANCA-associated vasculitis

**DOI:** 10.1093/ckj/sfag317

**Published:** 2026-10-09

**Authors:** Vanja Ivković, Sejin Park, Lauren Floyd, Yoon Seo Kwon, Annette Bruchfeld, Andreas Kronbichler, Jae Il Shin

**Affiliations:** Department of Health, Medicine and Caring Sciences, Linköping University, Linköping, Sweden; Department of Nephrology, Hypertension, Dialysis and Transplantation, University Hospital Center Zagreb, Zagreb, Croatia; Faculty of Health Studies, University of Rijeka, Rijeka, Croatia; Yonsei University College of Medicine, Seoul, Republic of Korea; Department of Renal Medicine, Lancashire Teaching Hospitals NHS Foundation Trust, Preston, Lancashire, UK; Yonsei University College of Medicine, Seoul, Republic of Korea; Department of Health, Medicine and Caring Sciences, Linköping University, Linköping, Sweden; Department of Renal Medicine, Karolinska University Hospital and CLINTEC Karolinska Institutet, Stockholm, Sweden; Department of Health, Medicine and Caring Sciences, Linköping University, Linköping, Sweden; Department of Internal Medicine IV, Nephrology and Hypertension, Medical University Innsbruck, Innsbruck, Austria; Department of Pediatrics, Yonsei University College of Medicine, Seoul, Korea; The Center for Medical Education Training and Professional Development in Yonsei-Donggok Medical Education Institute, Seoul, Korea; Severance Underwood Meta-research Center, Institute of Convergence Science, Yonsei University, Seoul, Korea; Section of Digestive Diseases, Department of Medicine, Yale School of Medicine, New Haven, CT, USA

**Keywords:** ANCA-associated vasculitis, evidence-based medicine, guidelines, quality, recommendations

## Abstract

**Objectives:**

We aimed to appraise 11 currently available guidelines for ANCA-associated vasculitis (AAV), identify potential gaps, and provide recommendations for improvement.

**Methods:**

The guidelines were appraised using the Appraisal of Guidelines for Research and Evaluation II (AGREE II) instrument consisting of 23 appraisal criteria/items across six domains (D). Four reviewers independently appraised guidelines and scaled domain scores were calculated.

**Results:**

The average score of all guidelines was 4.5 ± 0.8 out of 7.0. Agreement between the reviewers was excellent. British Society for Rheumatology 2025 guidelines scored highest (5.8/7.0), being the only guideline unanimously recommended for use. In ‘D1/Scope and purpose’, all guidelines achieved high scores. In ‘D2/Stakeholder involvement’, major gaps were identified in the inclusion of the views and preferences of the target population (2c), with few guidelines including patient representatives, addressing patient participation, or considering patient views. In ‘D3/Rigour of development’, all but two guidelines received high scores. Implementation of the procedure for updating the guidelines (3 h) and for external review (3 g) was addressed poorly. In ‘D4/Clarity of presentation’, all guidelines except one received a high score. ‘D5/Applicability’ had the lowest scores due to gaps in the criteria for monitoring/auditing guideline implementation (5d), resource/cost implications (5c), lack of advice/tools for implementation (5a), and inadequate addressing of facilitators/barriers in guideline application (5a). ‘D6/Editorial independence’ was addressed well.

**Conclusions:**

Several aspects of AAV guidelines could be improved, including the inclusion of patient views and preferences, the processes for translating guidelines into clinical practice, and clearer criteria for monitoring guideline implementation and uptake.

KEY LEARNING POINTS **What was known:**

- Clinical practice guidelines (CPG) provide much-needed guidance and recommendations on the management of diseases and are the cornerstone of modern clinical practice.
- Several CPGs have been published in ANCA-associated vasculitis (AAV) which are widely used. However, no study has evaluated the rigour and quality of these recommendations.

**This study adds:**

- We have evaluated 11 widely used CPGs for the management of AAV, scoring their quality using a validated instrument, the Appraisal of Guidelines for Research and Evaluation II (AGREE II), which comprises 23 appraisal criteria/items across six domains (D). Four raters have independently rated all CPGs to achieve a high level of objectivity.
- Our findings show that only one CPG was unanimously recommended for use and that several aspects of AAV guidelines could be improved.
- The most important weak links we identified are the inclusion of patient views and preferences, the processes for translating guidelines into clinical practice and clearer criteria for monitoring guideline implementation and uptake.
- Furthermore, we have provided concrete ideas and suggestions to address current gaps in AAV CPGs.

**Potential impact:**

- This is the first study which systematically evaluated the quality and rigour of CPGs in AAV.
- Our findings and suggestions might close quality gaps and inform the design of future CPGs for the management of AAV.

## INTRODUCTION

Clinical practice guidelines for the management of diseases are the cornerstone of modern, evidence-based medicine, aiming to optimize patient care [1]. These guidelines are created through complex multi-step processes involving multiple stakeholders, preferably including patient representatives, and combine a systematic review of evidence from trials and other studies with the experience and knowledge of key opinion leaders [1]. There is evidence that clinical practice guidelines improve both the quality of care and patient outcomes, leading to more cost-effective care and the optimal use of limited resources [2–8]. Anti-neutrophil cytoplasmic antibody-associated vasculitis (AAV) is no exception, especially given it is a heterogeneous and complex disease with variable clinical presentations and outcomes. As such, several guidelines for the management of this disease have been developed, mostly by international and national bodies [9]. These guidelines provide a much-needed summary of evidence and advice for the diagnosis and treatment of AAV, especially in the context of recent landmark randomized controlled trials that have changed the treatment landscape in this disease [10].

Given the reliance of clinical practice on guideline recommendations, several initiatives have been devised to evaluate their methodological quality, with Appraisal of Guidelines for Research and Evaluation II (AGREE II), a refinement of a previously used instrument, being the most widely used [11, 12]. AGREE II consists of 23 items across six domains and assesses multiple aspects of clinical practice guidelines, including quality of development and reporting and rigour of methodology [13]. The instrument was devised by an international consortium and remains the most comprehensively validated guideline assessment tool [11–14].

Guidelines, while not without flaws, have withstood the harsh test of time and have continually proven themselves to be an immensely valuable resource, a guiding star to physicians navigating decisions to ensure the best care for patients. However, it remains unknown whether there is indeed any *fault in our stars* (W. Shakespeare, Julius Caesar), as no study, to the best of our knowledge, has provided insights into the methodological quality of AAV guidelines. Methodological robustness is important as it ensures that all the steps have been taken to develop trustworthy and useful clinical practice guidelines. Thus, we aimed to critically appraise the currently used guidelines for the management of AAV using the AGREE II instrument, identify potential gaps and provide recommendations for their improvement.

## MATERIALS AND METHODS

### Inclusion of guidelines for assessment

We searched PubMed and Embase for guidelines for the management of AAV published in the last 10 years. Only English-language guidelines focusing specifically on the diagnosis and treatment of AAV were considered. In the next step, the most widely used AAV guidelines were selected for consensus among all authors and final inclusion in the review (Table 1) [15–25]. All selected guidelines fulfilled the 2011 Institute of Medicine definition of clinical practice guidelines. Consensus among all authors was required regarding whether the paper constitutes clinical practice guidelines under this definition [1].

**Table 1:** List of included guidelines/recommendations and their abbreviations.

Abbreviation	Publishing society or other body	Title of the guidelines	Journal, publication year
ACR/VF 2021	American College of Rheumatology and Vasculitis Foundation	2021 American College of Rheumatology/Vasculitis Foundation Guideline for the Management of Antineutrophil Cytoplasmic Antibody-Associated Vasculitis	Arthritis & Rheumatology, 2021
BSR 2025	British Society of Rheumatology	The 2025 British Society for Rheumatology management recommendations for ANCA-associated vasculitis	Rheumatology, 2025
CanVasc 2020	Canadian Network of Research on Vasculitides	CanVasc Consensus Recommendations for the Management of Antineutrophil Cytoplasm Antibody-associated Vasculitis: 2020 Update	Journal of Rheumatology, 2021
CanVasc 2022	Canadian Network of Research on Vasculitides	CanVasc consensus recommendations for the use of avacopan in antineutrophil cytoplasm antibody-associated vasculitis: 2022 addendum	Rheumatology, 2023
EULAR 2022	European Alliance of Associations for Rheumatology	EULAR recommendations for the management of ANCA-associated vasculitis: 2022 update	Annals of the Rheumatic Diseases, 2024
IRA 2024	Indian Rheumatology Association	Indian Rheumatology Association guidelines for the management of ANCA associated vasculitis	Autoimmunity Reviews, 2024
KDIGO 2024	Kidney Disease: Improving Global Outcomes	KDIGO 2024 Clinical Practice Guideline for the Management of Antineutrophil Cytoplasmic Antibody (ANCA)-Associated Vasculitis	Kidney International, 2024
MHLW 2023	Ministry of Health, Labour and Welfare, Japan	Clinical practice guidelines of the Japan Research Committee of the Ministry of Health, Labour, and Welfare for Intractable Vasculitis for the management of microscopic polyangiitis and granulomatosis with polyangiitis: The 2023 update—secondary publication	Modern Rheumatology, 2024
PANLAR 2024	Pan American League of Associations for Rheumatology	Pan American League of Associations for Rheumatology Guidelines for the treatment of ANCA-associated vasculitis	The Lancet Rheumatology, 2023
PNDS 2020	National Diagnostic and Care Protocol	French recommendations for the management of systemic necrotizing vasculitides (polyarteritis nodosa and ANCA-associated vasculitides)	Orphanet Journal of Rare Diseases, 2020
SEN 2025	Spanish Society of Nephrology	Recommendations for the diagnosis and treatment of anti-neutrophil cytoplasmic autoantibody associated vasculitis	Nefrologia, 2025

### Assessment of guidelines

AGREE II consists of 23 items grouped into six domains: (1) Scope and purpose, (2) Stakeholder involvement, (3) Rigour of development, (4) Clarity, (5) Applicability, and (6) Editorial independence. Guidelines were assessed independently by four appraisers (V.I., L.F., S.P., Y.S.K.; please find more information on appraisers in the following section). Each appraiser rated each item on a scale from 1 (strongly disagree) to 7 (strongly agree) and further provided an overall score from 1 (lowest possible quality) to 7 (highest possible quality) and his/her opinion on whether the guideline should be recommended for use (Yes; Yes, with modifications or No). Independent domain scores were calculated for each domain rather than aggregated into a single score, as per the instructions provided in AGREE II. Domain scores were calculated by summing up all the scores of individual items and a scaled domain score was calculated as the total percentage of the maximum possible score for that domain.

### Appraisers

All guidelines were assessed independently by four appraisers (V.I., L.F., S.P., Y.S.K.) who are well-versed in evidence-based decision-making and health care methodology. All four appraisers have carefully read and studied the AGREE II Instrument instructions (User’s Manual), the papers establishing AGREE II, and the training materials (the Overview Tutorial and the Practice Exercise). They have additionally reviewed other materials, including papers on the appraisal methodology and on the usefulness of the AGREE II tool, as well as several systematic reviews that used the tool. Two appraisers (L.F. and V.I.) are nephrologists with PhD with over 10 years of experience, focusing on clinical work and research in glomerular diseases and vasculitis. The other two appraisers (S.P. and Y.S.K.) are medical students who have additionally, as part of the activities of the Severance Underwood Meta-Research Center, received training in the application of the AGREE II instrument before participating as appraisers. The entire rating process was supervised and reviewed by JIS, a vasculitis expert and an affiliate of the Stanford meta-research centre and Director of the Severance Underwood Meta-Research Center.

### Quality of the guidelines

Quality was assessed for each domain using a two-tier system and classifying each domain as high quality (scaled domain score >70%) or low quality (scaled domain score ≤70%). While there is no recommended or established cut-off for quality classification, this threshold was suggested in the AGREE II instructions and a two-tiered system using this cut-off was shown to be the most widely used [26]. We assessed only the quality of domains and did not use a separate quality classification of each guideline, as is the accepted practice. However, each guideline was assessed in the last two questions (final assessment and recommendation of guideline for use) by each appraiser. The mean final assessment score for all guidelines was calculated across all appraisers, and votes were tallied for the recommendation to use. Quality of the guidelines was finally ranked using a hierarchical system with two levels: Level 1–Mean final assessment score and, if tied, Level 2–Recommendation of guidelines for use: number of votes for ‘Yes’, if tied then number of votes for ‘Yes, with modifications’, tied then number of votes for ‘No’.

### Statistical analysis

Continuous data were presented as mean and standard deviation. Categorical variables were presented as absolute and relative values. Inter-rater agreement was expressed as the intraclass correlation coefficient (ICC), presented as an absolute value with a 95% confidence interval (CI). While average measures ICC was the metric appropriate for our setting, we also calculated single-measures ICC. We used SPSS v. 27 (IBM Corp., USA) for all analyses.

## RESULTS

### Inter-rater agreement

Mean single measures ICC was 0.73 (95% CI 0.67–0.79; min to max: 0.60–0.83), indicating substantial agreement, while mean average measure ICC, which used the average score of the four reviewers, was 0.91 (95% CI 0.89–0.94; min to max: 0.86–0.95), indicating almost perfect agreement. Importantly, no guideline had an ICC (single- or average-measures) of less than 0.40 (the upper limit for fair agreement). In fact, no guideline had a single-measures ICC below 0.60 and an average-measures ICC below 0.86.

### Appraisal of guidelines

A total of 11 clinical practice guidelines published from 2010 to 2025 were appraised. Three guidelines were published by international and eight by national societies. All guidelines were in English, and one was additionally published in Spanish.

Three domains (‘Domain 2 Stakeholder involvement’, ‘Domain 3 Rigour of development’, and ‘Domain 5 Applicability’) received scores below 75%. Mean score in domain ‘Scope and Purpose’ was 89% ± 5%, for ‘Stakeholder Involvement’ 73% ± 11%, for ‘Rigour of Development’ 73% ± 10%, for ‘Clarity of Presentation’ 96% ± 4%, for ‘Applicability’ 54% ± 9%, and for ‘Editorial Independence’ 84% ± 14% (Fig. 1).

**Figure 1:**
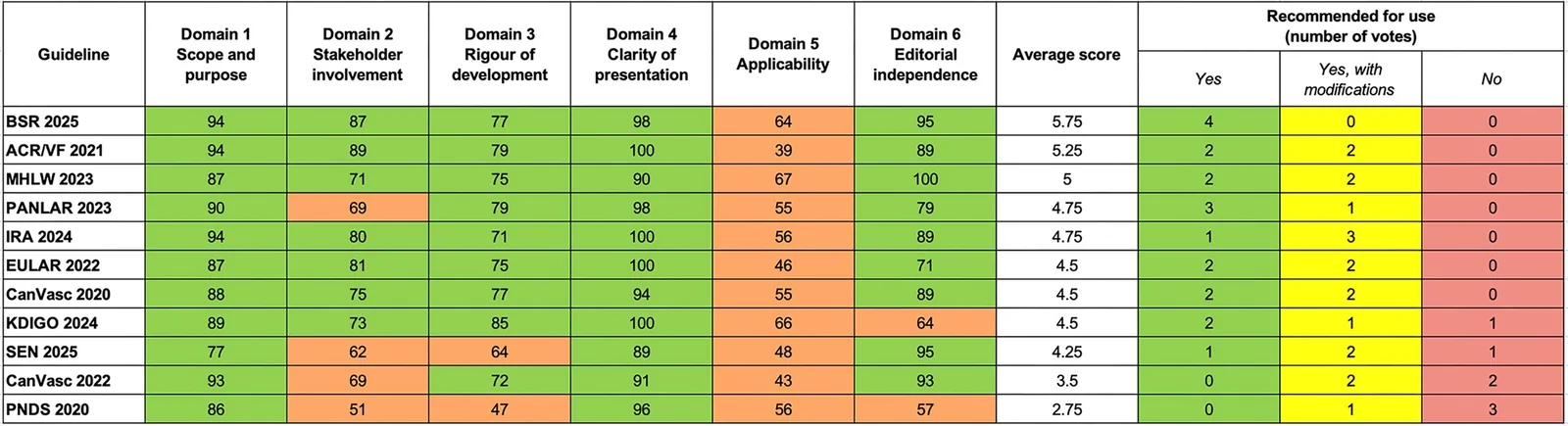
Scaled domain scores for all included guidelines and mean summary score and recommendation for use of all four reviewers. The British Society of Rheumatology 2025 guidelines have the highest score and are the only guidelines recommended by all four reviewers, while other guidelines are ranked differently based on their scores. ACR, American College of Rheumatology; BSR, British Society of Radiology; CanVasc, Canadian Network of Research on Vasculitides; EULAR, European Alliance of Associations for Rheumatology; IRA, Indian Rheumatology Association; KDIGO, kidney disease: improving global outcomes; MHLW, Ministry of Health, Labour and Welfare, Japan; PANLAR, Pan American League of Associations for Rheumatology; PNDS, National Diagnostic and Care Protocol; SEN, Spanish Society of Nephrology; VF, vasculitis foundation.

### Rating of guidelines across domains and items

In ‘Domain 1 Scope and purpose’, all guidelines achieved high scores (>70%) with the American College of Rheumatology/Vasculitis Foundation (ACR/VF) 2021, Indian Rheumatology Association (IRA 2024), and British Society of Rheumatology (BSR) 2025 scoring highest (94%) followed by Canadian Network of Research on Vasculitides (CanVasc) 2022 and Pan American League of Associations of Rheumatology (PANLAR) 2023 (both scoring >90%). Furthermore, all three questions in this section (1a. Overall objectives, 1b. Health questions and 1c. Population to apply) scored highly (≥6.0) and no major gaps were identified.

In ‘Domain 2 Stakeholder involvement’, scores for 2a. Identification of the guideline development group (5.4 ± 1.2) and 2c. Identification of users of guidelines, i.e. the physicians (6.3 ± 0.6) were good, while there was a major gap in 2b. Views and preferences of target population, i.e. the patients (3.8 ± 1.6), as few guidelines have fully included patient representatives, sufficiently addressed patient participation and taken into consideration patients’ preferences. Among the guidelines, the European Alliance of Associations for Rheumatology (EULAR) 2022 and ACR/VF 2021 had best assured patient inclusion.

Focusing on ‘Domain 3 Rigour of development’, which made the greatest contribution to overall quality, all guidelines except two had scores >70%. Spanish Society of Nephrology (SEN) 2024 guidelines had an overall score in this domain of 64.3% and National Diagnostic and Care Protocol (PNDS) 2020 guidelines had a score of 46.9%. Two main points in this domain which had the lowest scores were 3 h [updating procedure (3.1 ± 1.4)] and 3 g [review of guideline (3.3 ± 1.1)], with all other items having a score of 5 or higher. Among these, we emphasize the overall good scores for 3a [research methodology (6.0 ± 1.4)] and relatively good scores for 3c [strengths and limitations of evidence (4.9 ± 1.1)]. PNDS 2020 was the only guideline which had a low score (<3) for both items.

In ‘Domain 4 Clarity of presentation’, all guidelines, except SEN 2025 (89%), scored >90%. When looking at the three specific items (4a ‘Specific and unambiguous recommendations’, 4b ‘Different options for management’, and 4c ‘Identifiable key recommendations’), the guidelines all received a mean score >6.5 and there were no discernible gaps.

‘Domain 5 Applicability’ had the lowest scores. Further dissecting the reasons for the overall lowest score in ‘Applicability’, item 5d ‘Monitoring and/or auditing criteria’ was rated the lowest (3.3 ± 1.1), followed by 5c ‘Resource implications’ (3.8 ± 1.1), 5b ‘Guideline implementation advice and tools’ (3.9 ± 1.0), and 5a ‘Facilitators and barriers of application’ (4.1 ± 0.8). The ACR/VF 2021 guidelines had the lowest overall score in this domain (39.3%), while the Japanese Ministry of Health, Labour and Welfare (MHLW) 2023 (67.0%) and the Kidney Disease: Improving Global Outcomes (KDIGO) 2024 (66.1%) had the highest. All guidelines had a score lower than 70% and four guidelines had a score lower than 50%.

Lastly, ‘Domain 6 Editorial dependence’ was addressed well (score >70%) in all guidelines, except PNDS (57%) and KDIGO 2024 (64%). Looking at specific items, 6a ‘Views of funding body’ received a score of 5.6 ± 1.6 and 6b ‘Conflicts of interest’ 6.2 ± 0.6.

The summary of major strengths and gaps of guidelines in AAV is presented in Fig. 2. The recommendations for improvement of guidelines and addressing the most important identified gaps are presented in Table 2.

**Figure 2:**
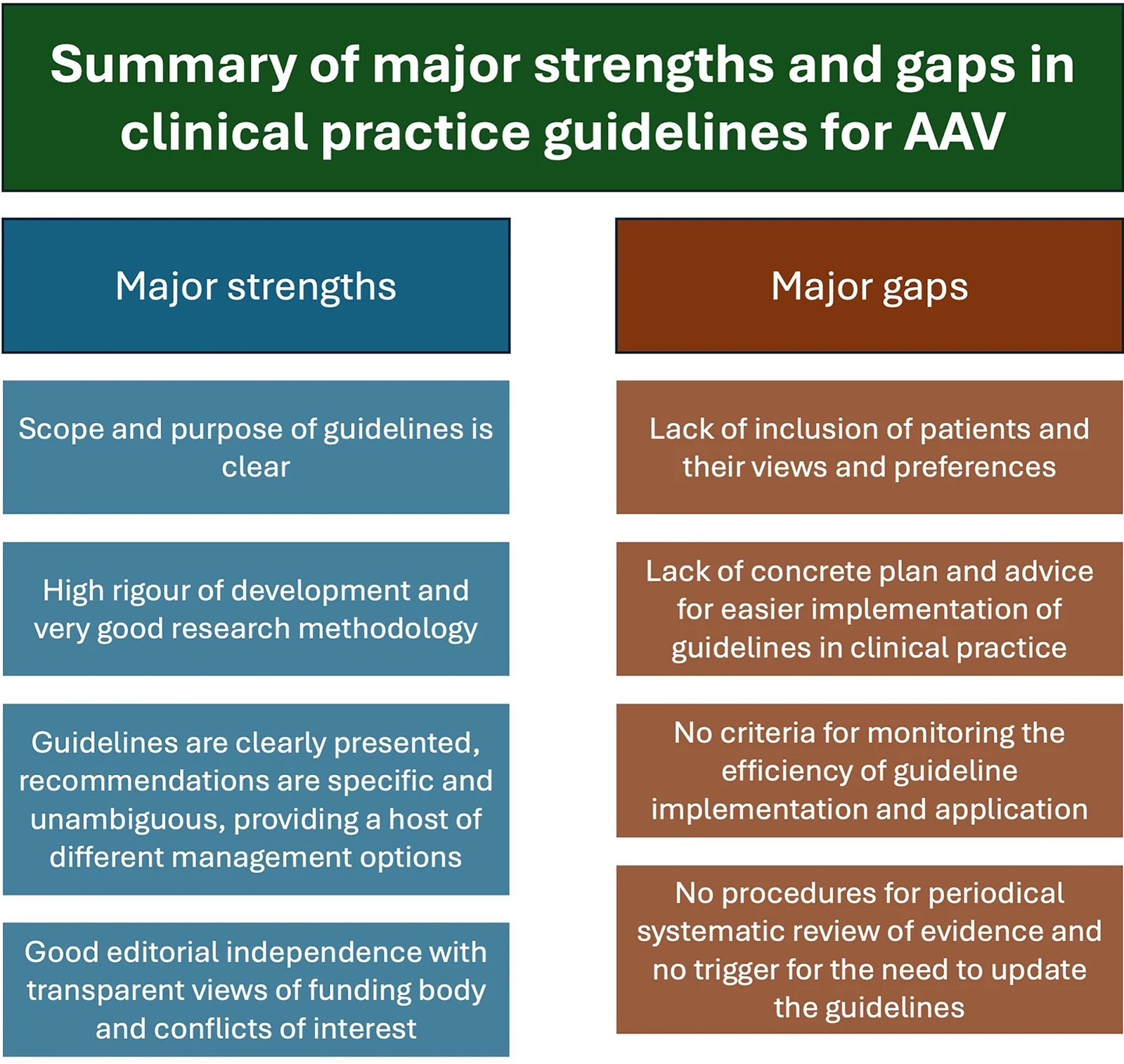
The summary of major strengths and gaps identified in clinical practice guidelines for ANCA-associated vasculitis (AAV).

**Table 2:** Significant gaps in the development of guidelines and recommendations, along with opportunities for enhancement.

Area	Major gaps	Suggestions for improvement
*Inclusion of patients’ views and their representatives*	Patient representatives are rarely fully included in the guideline development process	a) Patient representatives can be included in several different modalities, such as directly as individual panel members or collectively as part of reference groups or patient networks. b) If participating as panel members, they should be equal in all maters to other task force members. c) The timing of inclusion is critical; it is recommended that patient representatives participate at every stage of guideline development.
	Views and preferences of patients are not taken into consideration	a) Patient representatives should be allowed to freely provide comments on all aspects of guidelines during the whole process and especially on topics of greatest interest to them, such as treatment options. b) Alternatively, interviews can be done, or questionnaires can be provided to patient representatives to include their views and preferences. c) Patient representatives should be allowed to ask any questions or clarifications during guidelines development process.
*Methodology and planning*	Procedures for regular updating of guidelines are not implemented	a) Methodology and tools for scheduled literature searches should be developed to assure that recently published research is regularly checked and that major updates are collated for assessment when a new version or a partial update of the guidelines is needed. b) Pre-specified criteria should be developed to non-partially guide decisions when new guidelines or partial updates are needed. c) Periodical discussions on the need for an update should be done among the guideline development team.
	External review of guidelines is not always transparent	a) Guidelines should get a thorough, independent review from external experts. b) External review of guidelines should be made as transparent as possible.
*Implementation and applicability*	Lack of advice and tools on guideline implementation	a) Concrete advice should be provided on how to best implement and apply recommendations, especially those which might be complex or difficult to implement in daily practise. b) Where possible, tools such as risk calculators, mobile phone applications and patient portals, should be created and disseminated to ensure guidelines awareness and use.
	Lack of attention to resource implications, application in low resource setting and cost-benefit	a) Costs and cost-benefit (balance sheet of net benefits and incremental costs) should be considered when developing recommendations on diagnostics and therapies. b) In case of comparative safety and efficacy, diagnostic and therapeutic procedure with better cost-benefit ratio should be preferred and less costly alternatives should be presented whenever possible. c) Applicability of guidelines and availability of diagnostic and therapeutic procedures in low resource settings should be considered.
	Insufficient advice on facilitators of guideline implementation as well as on overcoming of potential barriers	a) Awareness of the guidelines should be raised through international and national societies, professional associations and social networks. b) Recommendations and overall outline of the guidelines should be made simple and easily understandable. c) Local adaptability of guidelines should be facilitated, and local versions should be endorsed, when appropriate.
	Lack of clear criteria for monitoring and auditing	a) Guidelines should provide clear criteria for monitoring and measuring their application. b) Clear criteria should be provided for monitoring fulfillment of goals and adherence to guidelines. c) Plan to assess and review the implementation and impact of guideline uptake.

### Overall assessment

Of the 11 included guidelines, seven were recommended for further use, with or without necessary modifications, by all raters and four received at least one negative vote (Fig. 1). Overall mean score for guideline quality was 4.5 ± 0.8, with BSR 2025 being the highest-ranked guideline.

## DISCUSSION

Our findings show that the most widely used clinical practice guidelines for the management of AAV are not all equal and several key gaps should be addressed to improve them. Importantly, none of the guidelines received an average score of 6 or 7, and only three (BSR 2025, ACR/VF 2021, and MHLW 2023) received an average score of 5 or higher, with only one guideline, BSR 2025, recommended for use by all four raters. While no guideline received four negative votes, four guidelines (KDIGO 2024, SEN 2025, CanVasc 2022, and PNDS 2020) have received at least one negative vote, with two guidelines (CanVasc 2022 and PNDS 2020) having two and three negative votes, respectively. The latter two are also the only guidelines that did not receive a clearly positive recommendation from any rater. When looking across domains, major gaps were identified in all included guidelines in ‘Domain 5 Applicability’ where no guideline scored a satisfactory (>70) score, ‘Domain 2 Stakeholder involvement’ where four guidelines received a score of 70 or less (SEN 2025, CanVasc 2022, PNDS 2020), ‘Domain 3 Rigour of development’ where two guidelines received a low score (SEN 2025 and PNDS 2020) and ‘Domain 6 Editorial independence’ where also two guidelines received a low score (KDIGO 2024 and PNDS 2025). In ‘Domain 1 Scope and purpose’ and ‘Domain 4 Clarity of presentation’, all the guidelines received a score of >70%. Our findings are in line with those previously reported across different areas of medicine and various diseases [27–37].

While the AGREE II instrument does not consider any domain more important than the other, several domains have been shown to carry more weight. ‘Domain 3 Rigour of development’ and ‘Domain 5 Applicability’ were shown to have the strongest influence on the two overall assessments, i.e. the final score on overall quality of each guideline assigned by the appraiser and whether the appraiser would recommend the guideline for use. Domain 3 has, moreover, been termed by some the strongest indicator of quality and critical importance [26, 27, 38, 39]. While ‘Rigour of development’ of AAV guidelines was generally acceptable (mean score 73%), there were major gaps when looking at individual items. Very few guidelines have adequately addressed the updating procedure, and most have not outlined future plans for updating. Similarly, information on external review by experts prior to publication was rarely, if ever, reported, and only a few guidelines provided details on any mode of external quality check. These omissions are important, as practically all modern guidelines undergo periodic updates, either as full revisions or as focused updates summarizing important developments since the publication of the previous version. Moreover, external peer review is the cornerstone of publication, and while all guidelines will certainly have undergone some degree of review, making this process transparent is crucial, as reviewers are expected to be critical of evidence characterization, recommendations and methodological rigour, thereby raising the quality of the final publication [40].

Stakeholder involvement (Domain 2) is very important and the gap in item 2b. Views and preferences of target population, i.e. the patients, are worrisome, as few guidelines have fully included patient representatives in development or sufficiently considered patient participation and preferences. Patients should have a voice in points in the guidelines of their interest, as their lived experience with the disease is invaluable for an integrative view. This ‘experiential evidence’ can complement, but sometimes also conflict with, the professional knowledge of clinicians and improve care experience and outcomes [41]. They should be included at every stage of development as panel members (individually or collectively), on equal footing with other task force members. During guideline development, they should be able to freely comment on all aspects, especially on the topics of greatest interest to them, such as treatment, and their views and preferences should be fully considered [41–43].

While updates of the guideline are needed, the frequency, extent and processes thereof are less clear [44]. This depends on the amount of new evidence and may vary with how propulsive the research in the field is at any time point [1, 44]. In the case of AAV, several key drugs have been shown to be efficacious in the last 5–10 years, and this is showcased in the fact that the three major international organizations (KDIGO, EULAR, and ACR/VF) have all published at least a partial update of their guidelines in the last 4 years. Most guidelines become obsolete after just under 6 years, so societies today often use a fixed update schedule of 2–5 years. However, a two-step process consisting of periodic literature checks to first identify potentially relevant new evidence and secondly decide if the evidence alters the validity of the current recommendations [44–46]. In fact, periodic assessment of guideline quality is needed to ensure that previously identified problems are addressed in the latest guidelines [30]. An optimal time frame is warranted to provide up-to-date guidelines while ensuring efficient use of resources, and it is important to strike a fine balance between the need to update the guidelines in response to new evidence and overly frequent updates, which may lead to confusion.

Guideline implementation, while important, is challenging and remains one of the weakest links in the guideline life cycle. Adherence to recommendations remains suboptimal across many areas of medicine [6, 47]. Ease of implementation should be planned during guideline development, addressed, and as much as possible, incorporated in the guidelines themselves, but the degree to which they will be adhered to and implemented in clinical practice largely depends on the primary users of the guidelines. A recent study showed a modest but clear gap between the evidence and clinical practice for eosinophilic granulomatosis with polyangiitis in Japan and recommended broader dissemination of updated guidelines and improved interdisciplinary collaboration to enhance patient care [48]. Low adherence, coupled with the fact that ‘Domain 5 Applicability’ is considered highly important yet is rarely adequately addressed in guidelines, led to the development of several tools, such as the GuideLine Implementability Assessment, which helps authors design optimal, tailored implementation strategies [49]. Moreover, the Guideline Implementation Success Assessment Tool (A-GIST), recently unveiled, is designed to comprehensively assess the extent of guideline implementation. A-GIST encompasses 20 items that integrate the perspectives of both clinicians and patients [50]. This tool could serve as a benchmark for assessing how well recommendations are implemented in clinical practice, a key step toward improving guideline adoption and patient outcomes.

Finally, while disclosure of conflict of interest has been identified as one of the key underreported areas in guidelines, AAV guidelines had high ‘Domain 6 Editorial Independence’ scores [30, 36]. This is very encouraging, as it shows commitment to transparency of key opinion leaders and authors in the field.

Alongside specific recommendations to improve AAV guidelines, there are also general recommendations which might be applied to all guidelines. Mandated registration of clinical practice guidelines, similar to that required for trials and systematic reviews, might improve transparency by enabling comparisons between proposed and final guideline reports, potentially impacting the quality of future guidelines [29]. Furthermore, while it is understandable that lack of space due to journal limitations may preclude the inclusion of all the necessary details in the main text, this information may be included in supplementary material. Lastly, journals might consider publishing AGREE II scores alongside the guidelines to showcase the objective assessment of their quality, thereby reflecting the strength of the guidelines’ recommendations [30].

This study has several strengths. We have included all widely used guidelines for the management of AAV. AGREE is the most widely used, systematic and structured tool for the assessment of clinical practice guidelines [12, 13, 51]. A total of four raters independently appraised all guidelines, which minimizes potential bias. Furthermore, interrater agreement was excellent overall, with ICCs for all guidelines at 0.86 or higher.

There are also some limitations. Firstly, we included only a selection of English-language guidelines based on the consensus of all authors, as our aim was to capture the most widely used AAV guidelines rather than all local or other published guidelines. However, the guidelines we selected for inclusion most likely represent a subset of methodologically higher-quality guidelines. Secondly, the AGREE II instrument is not without its faults and has been criticised for a confusing, time-consuming, and sometimes tedious scoring process, focused mainly on describing certain aspects of guidelines rather than verifying that they have been performed well [52]. However, the instrument has been developed through a meticulous process, has been verified in numerous studies and has the major strength of being subjected to thorough and systematic evaluation by the authors and users, helping it to continually evolve in the optimal direction [52]. While a low score in any domain of AGREE II does not necessarily mean that a guideline is not useful in clinical practice, it does imply that appropriate methodology and reporting may not have been followed [53]. Conversely, AGREE II does not guarantee clinical validity of the guidelines and clinical judgement should always be applied when selecting and using the appropriate guideline recommendations in clinical practice [54]. While many publications using AGREE II apply predefined cut-offs to classify domains by quality, there is no mandated or even recommended standardized score for doing so [26]. Studies used different strategies, valuing all or only some domains, or even combinations of domain scores; cut-offs ranging from 50% to 83% have been used as criteria for high quality. In our study, we used a relatively high, but probably the most widely accepted, cut-off of 70% to delineate high- from low-quality domains. The strength of evidence in rare diseases such as AAV is generally lower than in more common diseases, e.g. hypertension. While AGREE II is focused on methodological quality rather than the strength of evidence, for numerous previously reported reasons, rare diseases might achieve a lower AGREE II score [55, 56]. Even though their largest stakeholders are frequently the payor organizations, guidelines should primarily be targeted at practising clinicians, and the benefits they provide should be felt by patients. Guidelines should be adapted to these two target populations, potentially by including useful, practical clinical scenarios or by publishing secondary guidelines or summaries adapted for everyday clinical use or for patient use. Finally, we did not include raters of different specialties. Since AGREE II is a methodological tool, all appraisers, no matter the medical specialty, should come to similar conclusions on the methodological quality of the guidelines. However, guidelines are created by different professional societies and professionals of different specialties, and all guidelines do not have the same intended readership, so future studies might explore any potential bias caused by the selection of appraisers and any differences between e.g. nephrologists and rheumatologists in assessing the quality of vasculitis guidelines.

## CONCLUSIONS

While clinical practice guidelines in AAV have good development rigour and have been rated well in some other domains, there are key gaps that should be addressed, and only one guideline has been unanimously recommended for use without the need for modifications. Future guidelines should improve patient inclusion by fully integrating patient representatives, their views and preferences. Moreover, it is necessary to devise and incorporate processes and tools which will help translate guidelines into clinical practice and the criteria for monitoring the success of their implementation. These improvements are important to ensure AAV guidelines are not only methodologically robust but also patient-centred and clinically useful.

## Data Availability

The data underlying this article will be shared on reasonable request to the corresponding author.
